# Development of the pediatric narcolepsy patient-reported outcomes scale (PN-PROs)

**DOI:** 10.3389/frsle.2024.1379132

**Published:** 2024-04-26

**Authors:** Grace Y. Wang, Jennifer Worhach, Eric S. Zhou, Michael Strunc, Anne M. Morse, Judith A. Owens, Julie Flygare, Anna Revette, Lisa J. Meltzer, Kiran Maski

**Affiliations:** ^1^Department of Neurology, Boston Children's Hospital, Boston, MA, United States; ^2^Department of Psychosocial Oncology and Palliative Care, Dana Farber Cancer Institute, Boston, MA, United States; ^3^Department of Neurology, Children's Hospital of the King's Daughter, Norfolk, VA, United States; ^4^Department of Child Neurology and Pediatric Sleep Medicine, Janet Weis Children's Hospital, Geisinger Medical Center, Danville, PA, United States; ^5^Project Sleep, Los Angeles, CA, United States; ^6^Division of Population Sciences, Dana Farber Cancer Institute, Boston, MA, United States; ^7^Department of Pediatrics, National Jewish Health, Denver, CO, United States

**Keywords:** pediatrics, narcolepsy, qualitative development, patient-reported outcomes, sleep disorder, questionnaire, adolescent, children

## Abstract

**Background:**

Pediatric narcolepsy is a chronic neurological disorder that impacts the health and overall wellbeing of children and adolescents with the disease. Meaningful and regular assessment of symptom frequency and severity is important for the long-term management of narcolepsy and for optimizing quality of life. However, there is currently no patient-reported outcomes (PROs) measure developed from both patient and expert input that is designed specifically to assess the impact of pediatric narcolepsy on daily life and overall disease burden.

**Methods:**

We conducted a qualitative mixed-methods study to develop a novel patient-reported outcomes measure for pediatric narcolepsy patients. We created and refined a conceptual framework through literature review, semi-structured interviews with narcolepsy experts, and focus groups of children and adolescents with narcolepsy and their parents. Guided by the domains and facets identified in our conceptual framework and further literature review, we developed a PROs item bank. Our team further refined the item bank and classified items through team discussions and expert guidance. Content validity of the item pool was evaluated with expert review, readability analysis, and cognitive interviews with narcolepsy patients.

**Results:**

Through our processes, we developed a PROs item bank comprising two domains (narcolepsy symptoms and functional impairment) that contains 10 facets. The final item bank consists of 55 items, with 27 items representing five facets of narcolepsy symptoms (behavior, cataplexy, cognitive difficulties, sleep quality, and sleepiness) and 28 items representing five facets of functional impairment (bothered by/worried about symptom, cognitive/academic, global functioning, safety, and social).

**Conclusion:**

We developed items for the Pediatric Narcolepsy Patient-Reported Outcomes (PN-PROs) measure that incorporates expert insight, published literature, and testimony from children/adolescents living with narcolepsy and their families. Upon completion of the psychometric testing and content validation process, we believe the PN-PROs will provide a useful longitudinal measure of disease control and standardize outcome assessments in clinical practice and research studies.

## 1 Introduction

Pediatric narcolepsy is a debilitating chronic neurological disorder that is characterized by excessive daytime sleepiness, cataplexy, hallucinations, sleep paralysis, and disrupted nighttime sleep (American Academy of Sleep Medicine, 2014). There are two subtypes of narcolepsy: Narcolepsy type 1, which is caused by the near-complete loss of orexin neurons in the lateral hypothalamus (American Academy of Sleep Medicine, 2014) and narcolepsy type 2, which has unknown etiology (Mignot et al., 2002). More than 50% of people with narcolepsy report symptom onset before the age of 18 years (Thorpy and Krieger, 2014). Uncontrolled narcolepsy symptoms can lead to psychosocial issues, as children and adolescents with narcolepsy may not be able to meet academic or work-related demands (Rocca et al., 2016; Plazzi et al., 2018; Graef et al., 2020). Narcolepsy currently has no defined treatment, resulting in a disorder that has a significant and lifelong impact on the psychosocial health and quality of life of those diagnosed (Rocca et al., 2016). Consequently, there is a need to develop and validate a narcolepsy outcomes scale that incorporates pediatric language and experiences to capture disease burden and outcomes.

Patient-reported outcome measures are a way to incorporate the thoughts of children and adolescents with narcolepsy into a measure that evaluates disease difficulties, determines treatment efficacy, and guides future drug development. The Food and Drug Administration (FDA) defines patient-reported outcomes (PROs) as “any report of the status of a patient's health condition that comes directly from the patient without interpretation of the patient's response by a clinician or anyone else” (Higgins et al., 2023). PROs are important to help understand patient perspectives regarding their lived experience with treatment benefits and harms, as well as disease impact on their daily functions. As pediatric narcolepsy is a condition with few externally observable symptoms, PROs are an especially important way to measure disease control and patient wellbeing as well as assess treatment needs.

Meaningful and regular assessment of symptom frequency and severity is important for the long-term management of the disease, with several measure utilized for pediatric narcolepsy symptoms. Currently, the Epworth Sleepiness Scale for Children and Adolescents (ESS-CHAD; Janssen et al., 2017) and the Pediatric Daytime Sleepiness Scale (Drake et al., 2003) are the most commonly used scales used clinically and in research to assess narcolepsy symptoms; however, these scales focus solely on daytime sleepiness and no other narcolepsy symptoms that contribute to disease burden. The Ulanlinna Narcolepsy Scale includes assessments of excessive daytime sleepiness, cataplexy, and sleep onset latency (Hublin et al., 1994) and has been used in pediatric narcolepsy clinical trials (Lecendreux et al., 2017; Dauvilliers et al., 2023). However, the Ulanlinna Narcolepsy Scale was developed for adult narcolepsy type 1 patients and has not been validated in pediatric narcolepsy populations.

More recently, pediatric narcolepsy specific scales have been developed and validated. A pediatric narcolepsy quality of life scale (NARQoL-21) was developed and validated in a Swedish population (Chaplin et al., 2017) and a Chinese population (Li et al., 2021). This 21-item measure includes two factors: Psychosocial and Future Outlook. Although the development process included youth with narcolepsy, the questions do not focus on direct attribution of health related quality of life (HRQoL) to narcolepsy symptoms, nor does it include HRQoL related to cataplexy. A Pediatric Narcolepsy Severity Scale (P-NSS) was developed and validated for Chinese patients aged 8–18 years, with items focused on symptom severity after the diagnosis of narcolepsy type 1 (Li et al., 2023). The domains align with narcolepsy symptoms, including sleepiness, cataplexy, sleep-related hallucinations, sleep paralysis, and hyperkinetic behavior/automation. This scale has yet to be translated into English and validated in populations outside of China. A similar titled survey, the Pediatric Narcolepsy Severity Scale (NSS-P), was developed and validated in France (Barateau et al., 2021). Although available in English, the NSS-P has only been validated in a French speaking population. While the NSS-P shows excellent psychometric properties in children ≥10-year of age and adolescents, it was developed from a “top down” approach, with experts in narcolepsy adapting an adult narcolepsy scale (Dauvilliers et al., 2020). The team did not conduct pediatric focus groups or formally conduct interviews to assess understanding of the questions in the pediatric population. Arguably, without patient input in the development phase, a patient outcomes scale neglects identifying important patient needs and values and may miss an opportunity to engage respondents to enhance accuracy and cooperation.

To ensure transparency in measurement development, as well ensure methodological rigor and reproducibility, we present in this paper the rigorous, mixed-method process completed for the development of the novel Pediatric Narcolepsy Patient-Reported Outcomes (PN-PROs) measure. Unlike most previous measures, this included a comprehensive literature review to both conceptualize the framework and elicit items from existing measures, as well as interviews with patients, parents, and narcolepsy experts at each step of the process to ensure the final measure is relevant and meaningful.

The goal of developing the PN-PROs was to expand previous work to integrate narcolepsy core and related symptoms, as well as broader health-related quality of life outcomes. We anticipate the final validated product will allow clinicians and researchers to ensure that treatments are effective for all aspects of a patient's quality of life, not only their disease symptoms.

A subsequent paper will provide the outcomes for the final step of the measurement development process, namely psychometric testing in a large population of pediatric patients with narcolepsy or obstructive sleep apnea.

## 2 Methods

This study was approved by the Institutional Review Board of Boston Children's Hospital (IRB-P00029346). All participants over the age of 18 years old provided informed consent to the research procedures. All participants under the age of 18 provided informed assent to the research procedures and their parents or guardians provided informed consent.

Qualitative development of the Pediatric Narcolepsy Patient-Reported Outcomes Scale followed methods established by previous PROs measures (DeWalt et al., 2007; Lasch et al., 2010; Bevans et al., 2019). We first created a preliminary conceptual framework based on a systematic literature review and refined the framework through expert, child, and parent interviews. We then used our conceptual framework to inform item bank development. The initial items were selected from previous measures and further refined through expert review and cognitive interviews with children and adolescents with narcolepsy.

### 2.1 Conceptual framework development

#### 2.1.1 Step 1: systematic literature review

We conducted a systematic literature review to identify child- and parent-reported symptoms of narcolepsy (e.g., sleepiness, cataplexy, dreams/nightmares, weight, precocious puberty, fatigue, brain fog, attention, memory, hyperactivity, and behavioral problems) and functional effects of narcolepsy symptoms (e.g., school performance, quality of life, driving safety, and internalizing symptoms). The search was conducted in MEDLINE, Cochrane Controlled Trials, and Embase (see Supplementary Figure 1 for a sample search in Embase), with only English-language results reviewed. Results were reviewed by two investigators to create the initial framework, including domains, facets (conceptually distinct categories), and sub-facets (sub-categories).

#### 2.1.2 Step 2: expert interviews

We refined our conceptual framework through semi-structured interviews conducted with three pediatric narcolepsy experts (EZ, MS, AM) outside of the study team. The experts had a minimum of two peer-reviewed publications in pediatric narcolepsy and actively saw pediatric narcolepsy patients in their clinical practice. During the interviews, the experts were asked open-ended questions about common language children and adolescents with narcolepsy and their parents use to report symptoms of concerns, common consequences of untreated/undertreated symptoms, symptoms that change with treatment, and outcome measures.

#### 2.1.3 Step 3: child and parent focus groups

We refined our PN-PROs conceptual framework further through focus group interviews with 14 children and adolescents 10–18 years old with a diagnosis of narcolepsy and their parents (*n* = 14). We recruited participants through advertisements in sleep clinics at Boston Children's Hospital and advertisements on narcolepsy patient support group websites (i.e., Wake Up Narcolepsy, Project Sleep, Narcolepsy Network, Hypersomnia Foundation, etc.). Narcolepsy diagnoses were confirmed through a medical record review, using International Classification of Sleep Disorders version 3 (ICSD-3) criteria, or a signed letter from the participant's physician confirming the diagnosis.

Patient and parent interviews ensured that the conceptual framework reflected the lived experiences of the population that will be using the measure as a PRO. Before the interviews, participants completed an online survey through REDCap, with both quantitative and open-ended questions about narcolepsy symptoms, including symptom effects and importance of symptoms for quality of life and daily functioning. We asked children about their own symptoms, and parents were asked about their child's symptoms (proxy-report).

### 2.2 Item bank development/identification

#### 2.2.1 Step 4: identification of initial item pool

The previously described systematic literature review was also used to identify child- or parent-report measures that included items matching the identified facets in our conceptual framework. Once measures that captured narcolepsy symptoms or functional impairments were identified, individual items were extracted. Through regular team discussions, consensus was reached about items that were considered redundant or not relevant to the conceptual framework. The remaining items were binned by the study team, with individual items assigned a domain (symptom or function), facet (e.g., behavior), and sub-facet (e.g., disorganization). Additional items considered redundant were also removed (winnowed). If needed, items were re-written to fit within a 2-week recall period. As some symptoms (e.g., cataplexy) and functional impairment experiences may not occur within a 2-week period, other time frames (i.e., 1- or 3-month recall) were adopted.

#### 2.2.2 Step 5: expert review, cognitive interviews, readability analysis

The items identified through the systematic literature review were reviewed by outside experts with expertise in pediatric narcolepsy (JO, EZ), as well as an advocate for people with narcolepsy (JF). One researcher (AR) then conducted cognitive interviews to identify any problems with language used, item comprehension, recall, and other cognitive processes related to our developed questions (Irwin et al., 2009). We recruited 11 children and adolescents ages 8–17 years with narcolepsy to complete cognitive interviews of our drafted survey questions. Interviews were conducted using the HIPAA compliant Zoom teleconferencing program. Before the interview, the participants completed a survey with the items from the expert review. We asked participants questions about their comprehension of each of the survey questions and obtained their overall feedback on the survey. Questions were then revised through re-wording and re-ordering item presentation. The finalized items were reviewed for readability by calculating the Flesch-Kincaid Grade Level using Microsoft Word.

### 2.3 Data analysis

We recorded and transcribed all expert interviews, focus groups, and cognitive interviews. The team used thematic analysis to explore meaningful and relevant experiences with pediatric narcolepsy (Clarke, 2013). Through the review of all transcripts, we developed a codebook for individual sub-groups (experts, parents, and patients). We iteratively updated codebooks to include both deductive and inductive codes, with deductive codes allowing for the exploration and analysis of consistent domains across sub-groups (Green, 2014). Then, we applied finalized codebooks to all applicable transcripts. Once coding was complete, we summarized, reviewed, and discussed the coded data as a team (KM, AR, MB, LM). Analysis focused on identifying key patterns, contexts, and dimensions of the narcolepsy experience both within and between subgroups. Each stage of the coding and analysis was shared and discussed with the research team. These methods were enhanced using the NVIVO qualitative data management software program (QSR International, version 10).

For the cognitive interviews, we summarized each of them and then aggregated summaries from each round of interviews in a matrix in excel. The team reviewed and discussed feedback within and across each interview to determine if any appropriate changes would be made to specific items.

## 3 Results

### 3.1 Participant demographics

In our focus groups interviews to develop the initial item bank, 14 children and adolescents 10–18 years old (mean age 13.8 ± 2.3) with a diagnosis of either narcolepsy type 1 (*n* = 11) or narcolepsy type 2 (*n* = 3) and their parents (*n* = 14) participated. 42.9% of our focus group sample were female and 21.4% male gender. The remainder of the sample preferred not to disclose their gender. Pediatric participants were 35.7% Caucasian and 14.3% Black. The remainder preferred not to disclose their race.

Our cognitive interview sample consisted of 11 children/adolescents with narcolepsy type 1. The pediatric participants had a mean age of 13.5 years (*SD* = 3.4). 54.5% of this group were females and 45.5% were males. Race distribution was similar to our focus groups with 36.4% Caucasian and 18.2% Black. The remainder of the sample preferred to not disclose their race.

### 3.2 PN-PROs item bank

#### 3.2.1 Step 1: conceptual framework

**Figure 1:**
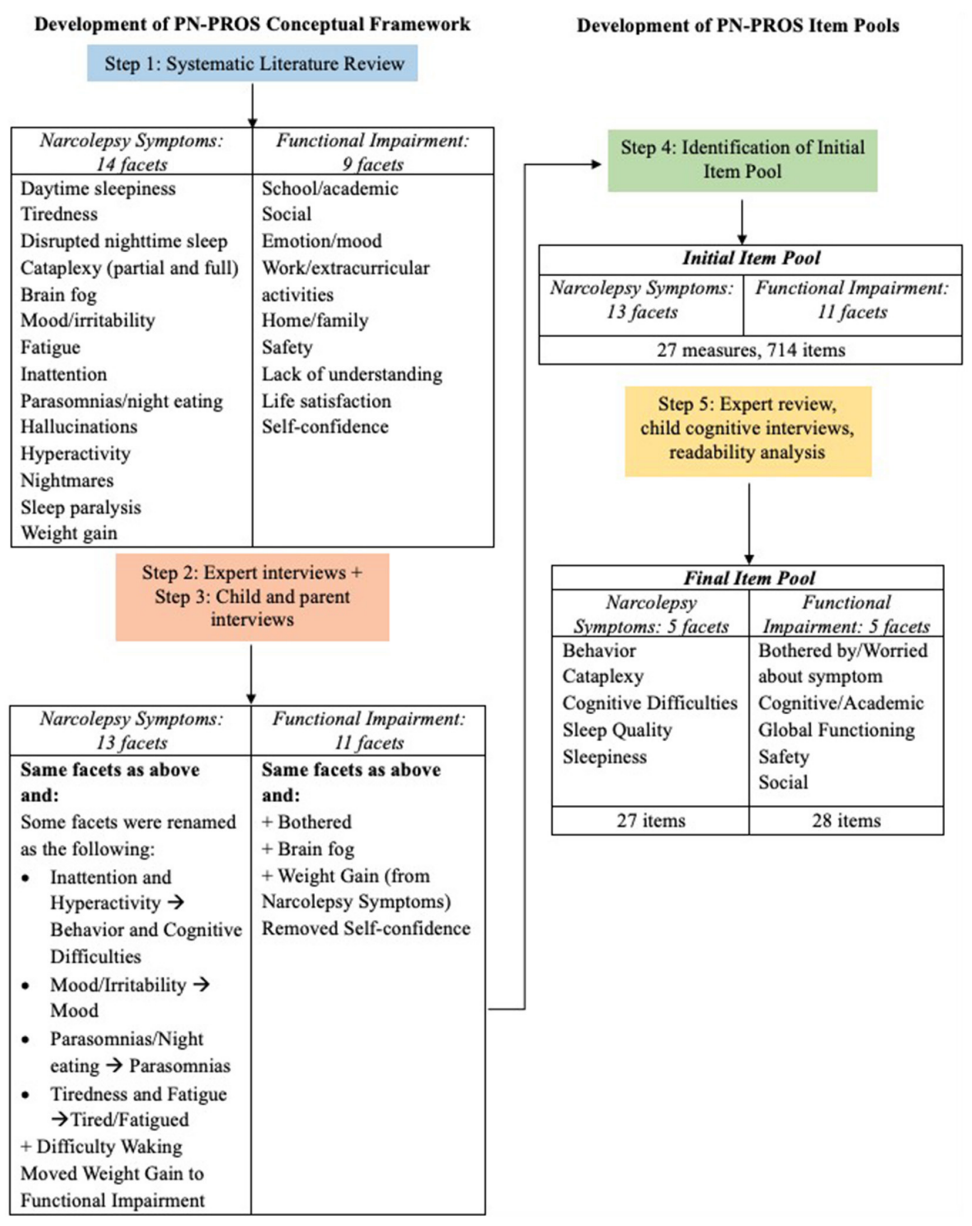
PN-PROs narcolepsy symptom and functional impairment conceptual framework and item pool development process.

Figure 1 shows a schematic of the steps taken to develop our conceptual framework and final item pool. Based on the first systematic literature review, we initially identified two domains: Narcolepsy symptoms and functional impairment. Within the narcolepsy symptoms domain, 14 conceptually distinct subcategories (facets) were identified, and within the Functional Impairment Domain, nine facets were identified. The facets acted as our preliminary conceptual framework to guide our expert and focus group interviews.

#### 3.2.2 Steps 2 and 3: expert interviews/child and parent focus groups

Through interviews with experts, children and adolescents with narcolepsy, and parents, the conceptual framework was further refined. Experts provided input on the facets and sub-facets, while results from the patient/parent focus groups identified the frequency of symptoms (both the most common and least commonly experienced concerns). Table 1 includes the frequency of narcolepsy symptoms mentioned during the focus groups, and examples of specific comments given during the interview portion with the children/adolescents and their parents. The final framework included 13 symptom facets and 11 functional impairment facets, with certain facets renamed, added, or removed from the original facets identified in the literature review (Figure 1).

**Table 1:** The number of participants that stated they were concerned or had not experienced a certain narcolepsy symptom of disease concern during the focus groups.

**Symptoms**	**High rank/top 5 rank**	**Not experienced/not bothersome**	**Other comment**
Brain fog	Parent: *n* = 8	Parent: *n* = 2	“When I get brain fog, I could want to do something, and then can just forget.”
	Child: *n* = 6	Child: *n* = 1	
Daytime sleepiness	Parent: *n* = 11	Parent: *n* = 1	“It affects her but it doesn't upset her.”
	Child: *n* = 12		
Disrupted nighttime sleep	Parent: *n* = 2 Child: *n* = 8		“Like it's very frustrating when I wake up in the middle of the night once every hour and it takes like 45 min to fall back asleep. It just gets really frustrating, because I feel like it's going to lead me to have an unproductive day.”
			“I'll wake up like three, four, five times, like during the night. And I'll either check my phone or I'll just like flip over and try to fall back asleep.”
Fatigue	Parent: *n* = 7		“The one symptom that I just cannot fight.”
	Child: *n* = 11		
Cataplexy	Parent: *n* = 6	Parent: *n* = 3	“It's the most visible.”
	Child: *n* = 7	Child: *n* = 2	“It's just for her, it's this constant uncertainty.”
			“I don't take my cataplexy too seriously.”
			“It doesn't really bother me a whole lot.”
Hallucinations that occur when falling asleep or waking up	Parent: *n* = 1 Child: *n* = 2	Child: *n* = 1	“I get sleep hallucinations and stuff, and they're terrifying but they don't really bother me. Like they're not inconvenient.”
			
Hyperactivity	Parent: *n* = 2 Child: *n* = 2	Parent: *n* = 2	“Sometimes she's just like a train. You can't shut her off.”
			
Inattention	Child: *n* = 6	Parent: *n* = 1	“It's difficult to even focus or pay attention.”
Irritability	Parent: *n* = 5		“Sometimes I get in a bad mood and I don't really know why.”
	Child: *n* = 8		
Dreams/nightmares	Parent: *n* = 2	Parent: *n* = 1	“I get really weird dreams….but it's not really that bad.”
	Child: *n* = 2	Child: *n* = 1	
Parasomnias/night eating	Parent: *n* = 2	Parent: *n* = 1	“I do sleep talk, but I don't sleep walk.”
	Child: *n* = 2	Child: *n* = 2	“I'll just wake up in the middle of the night and start talking to a person who is not there.”
			“He's never not either eating or walking around any night.”
Sleep paralysis	Parent: *n* = 3 Child: *n* = 2	Child: *n* = 2	“I feel like sleep paralysis is a major thing…I feel like I can't move…so it's kind of scary.”
			
Weight gain	Parent: *n* = 2	Parent: *n* = 1	“He's gained a lot of weight, which also doesn't help his self-esteem.”
	Child: *n* = 3	Child: *n* = 1	“They're classifying it as weight gain, but I don't think that's the issue.”

All frequencies for each symptom were divided into the parent sample and child sample. Quotes from the focus groups were included for certain symptoms.

#### 3.2.3 Steps 4 and 5: identification of initial item pool/expert review, cognitive interviews, readability analysis

The initial item pool included 27 existing measures and 714 items (13 narcolepsy symptom facets and 11 functional impairment facets). With redundant or irrelevant items removed, 337 items went through the bin-winnow, with an additional 129 items removed. The experts reviewed the resulting 208 items, and the feedback provided resulted in an additional 75 items removed. The 133 items were administered during the cognitive interviews with patients and parents with their feedback resulting in a further reduction of the item pool. The reading level analysis of the final 55 items using Flesch-Kincaid Grade Level equivalent available through Microsoft Word software is at a 3rd grade level.

#### 3.2.4 Final item bank

The final Pediatric Narcolepsy Patient-Reported Outcomes item bank (which is currently undergoing field testing) includes a total of 55 items. Within narcolepsy symptoms, there are 27 items that reflect five facets (behavior, cataplexy, cognitive difficulties, sleep quality, and sleepiness). These 27 items address the direct impact of narcolepsy symptoms on quality of life and outcomes (e.g., “I avoided activities or playing sports because of my narcolepsy symptoms,” “I got hurt or nearly hurt because of my cataplexy,” “I did not enjoy activities because of my narcolepsy symptoms in the last 2 weeks”). The other 28 items represent the functional impairment domain, which reflects five facets (bothered by/worried about symptom, cognitive/academic, global functioning, safety, and social). These 28 functional items are based on behavior, social function, and executive functioning problems commonly reported in narcolepsy patients (e.g., “I had a hard time finishing things I had to do,” “I was disorganized,” “I got annoyed easily,” “I have been forgetful,” “I had problems paying attention”).

## 4 Discussion

Patient-reported outcome measures are essential for both narcolepsy clinical care and research, going beyond disease symptom frequency and severity, and identifying disease- or treatment-related quality of life outcomes. In a clinical setting, PROs increase the opportunity for patient-centered care and facilitate communication and shared decision making between providers and patients. Additionally, PROs can help healthcare providers identify and optimize patient treatment needs. For research, validated PROs can standardize outcomes necessary for comparative effectiveness studies and highlight areas of unmet needs among people with narcolepsy.

In the paper we described the best practices, rigorous process we utilized to develop the Pediatric Narcolepsy Patient-Reported Outcomes Scale (PN-PROs). Notably, in addition to a systematic literature review, we collected expert input from physicians, a psychologist, a patient advocate, as well as children and adolescents with narcolepsy and their parents. Stakeholder input captures the patients' lived experience with narcolepsy and their perspectives on all aspects of quality of life, including symptom frequency, functional abilities, and psychosocial needs, and not just what symptoms are important to professionals in the clinical setting.

The final PN-PROs item bank includes 55 items for either self-report or parent/guardian proxy-reporting. While other recent measures [i.e., NARQoL-21 (Chaplin et al., 2017), P-NSS (Li et al., 2023), and NSS-P (Barateau et al., 2021)] have also been developed to measure symptoms and/or quality of life in patients with pediatric narcolepsy, each measure has limitations in the development (e.g., using a “top down” approach, not including key stakeholders) or the content (e.g., focusing only on narcolepsy symptoms). Once validated, we believe the PN-PROs will be a useful tool for clinicians and researchers, providing a narcolepsy-specific PRO measured that is inclusive of patient, caregiver, and provider input, assesses disease- and subjective-symptom frequency, and captures quality-of-life outcomes. This will allow clinicians and researchers to ensure that treatments are effective for all aspects of a patient's quality of life, not only their disease symptoms.

A previous survey of over 1,500 adults with narcolepsy found subjective symptoms, such as cognitive difficulties, emotional problems/irritability, and nocturnal sleep disturbances were more impactful on daily life and wellbeing than well-known narcolepsy symptoms of sleep paralysis and sleep-related hallucinations (Maski et al., 2017). Similarly in our study, the majority of pediatric focus group participants identified daytime sleepiness, fatigue, disrupted nighttime sleep, and irritability as the most problematic symptoms. Such insight drove us to broaden the PN-PROs to not only include assessments of core narcolepsy symptoms and impact on daily life, but also include associated symptoms and co-morbidities, as seen in the functional impairment domain. Notably, other instruments available for pediatric populations such as the Ulanlinna Narcolepsy Scale (Hublin et al., 1994) and NSS-P (Chaplin et al., 2017) do not include symptom concerns relating to fatigue and irritability. Given its more holistic view of disease burden, we believe the PN-PROS will be a useful adjunct assessment of disease burden along with currently available surveys focused symptom severity.

Our qualitative approach for developing the PN-PROs item bank was conceptually based and included input from key stakeholders. We conducted a time-consuming, rigorous process that followed best practices for PROs development (Irwin et al., 2009; Clarke, 2013; Green, 2014). However, there were limitations with our process. First, while the final item pool represents a wide variety of pediatric narcolepsy outcomes, it is not exhaustive. For example, the final PN-PROs does not assess environmental conditions, non-disease specific stressors, or the presence of diagnosed mood disorders that could influence symptom severity reporting. Second, the PN-PROs is designed to assess symptoms for both children and adolescents with NT1 and NT2. However, the majority of our participants were diagnosed with NT1 and most participants were adolescents. Thus, some items may not be applicable for younger cohorts and NT2 patients (such as cataplexy), and we may have missed items applicable to these groups. While our sample sizes may seem small compared to large clinical trials, we followed qualitative research guidelines and ensured that we reached saturation in our interviews and cognitive testing. Concerns about restricted sample characteristics will be further studied in our multi-site validation phase, in which we will conduct sensitivity analyses to determine the influences of age and treatments on PN-PROS scores. Lastly, we interviewed narcolepsy patients that were already diagnosed and receiving treatment, including medications, which could have influenced how participants perceived the importance of their symptoms. However, the PN-PROs was not developed to be a diagnostic tool, but rather a measure of patient-reported outcomes for youth already diagnosed with narcolepsy to ensure treatment is meeting not only disease symptoms, but broader functioning.

### 4.1 Implications and future directions

We have developed a new measure to assess pediatric narcolepsy outcomes using best practices for PROS. We foresee the PN-PROs being useful in clinical and research settings because (1) it assesses narcolepsy symptoms using scenarios familiar and language understandable to children and adolescents and (2) it includes domains indirectly impacted by narcolepsy symptoms not typically assessed in narcolepsy symptom surveys such as brain fog, executive function, academic performance, and social isolation. Validation and reliability testing is being completed in a diverse, North American sample of pediatric narcolepsy patients (9–17 years) across five sites. We will include a comparison group of children/adolescents with obstructive sleep apnea to determine discriminative validity of the instrument. We are also studying associations between PN-PROs with other validated quality of life measures to test our claim that this instrument meaningfully measures disease burden. Upon completion of the psychometric testing, we believe the PN-PROs will be useful instrument to provide a measure of disease control and wellbeing, highlighting areas that are important to the patient population living with narcolepsy that may benefit from psychological, academic, and/or social supports, and standardizing outcomes in both clinical practice and research studies.

## Data availability statement

The datasets presented in this article are not readily available because they contain patient health information that could compromise the privacy of research participants. Requests to access the datasets should be directed to kiran.maski@childrens.harvard.edu.

## Ethics statement

The studies involving humans were approved by Institutional Review Board of Boston Children's Hospital. The studies were conducted in accordance with the local legislation and institutional requirements. Written informed consent for participation in this study was provided by the participants' legal guardians/next of kin.

## Author contributions

GW: Formal analysis, Writing – original draft, Writing – review & editing. JW: Investigation, Project administration, Writing – review & editing. EZ: Writing – review & editing, Supervision. MS: Supervision, Writing – review & editing. AM: Supervision, Writing – review & editing. JO: Supervision, Writing – review & editing. JF: Supervision, Writing – review & editing. AR: Writing – review & editing, Formal analysis, Investigation, Methodology. LM: Methodology, Writing – review & editing, Conceptualization, Funding acquisition, Project administration, Supervision, Writing – original draft. KM: Conceptualization, Funding acquisition, Methodology, Project administration, Supervision, Writing – original draft, Writing – review & editing.

## Supplementary material

Supplementary Figure 1 Search terms used in the literature search.
