# Supplementary figures and images for: Development of the pediatric narcolepsy patient-reported outcomes scale (PN-PROs)
Source: Front Sleep. 2024 Apr 26;3:1379132. doi: 10.3389/frsle.2024.1379132 (PMC12713932; doi:10.3389/frsle.2024.1379132)

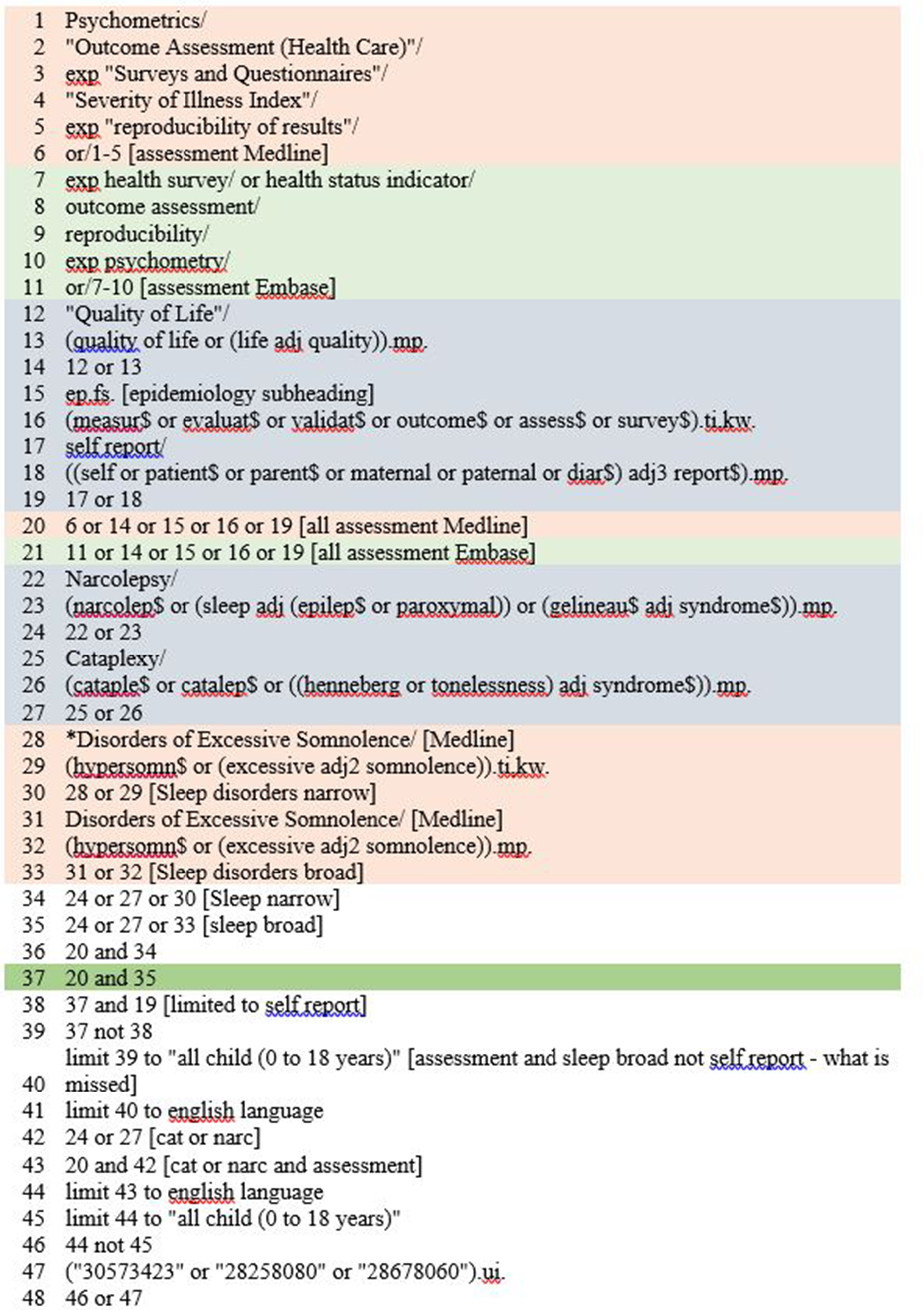

Supplement: Supplementary Figure 1 — Search terms used in the literature search. [file Image_1.JPEG]
